# Using compositionality to understand parts in whole objects

**DOI:** 10.1111/ejn.15746

**Published:** 2022-07-20

**Authors:** S. P. Arun

**Affiliations:** ^1^ Centre for Neuroscience Indian Institute of Science Bangalore India

**Keywords:** holistic processing, object recognition, object vision, visual perception

## Abstract

A fundamental question for any visual system is whether its image representation can be understood in terms of its components. Decomposing any image into components is challenging because there are many possible decompositions with no common dictionary, and enumerating the components leads to a combinatorial explosion. Even in perception, many objects are readily seen as containing parts, but there are many exceptions. These exceptions include objects that are not perceived as containing parts, properties like symmetry that cannot be localized to any single part and special categories like words and faces whose perception is widely believed to be holistic.

Here, I describe a novel approach we have used to address these issues and evaluate compositionality at the behavioural and neural levels. The key design principle is to create a large number of objects by combining a small number of pre‐defined components in all possible ways. This allows for building component‐based models that explain neural and behavioural responses to whole objects using a combination of these components. Importantly, any systematic error in model fits can be used to detect the presence of emergent or holistic properties. Using this approach, we have found that whole object representations are surprisingly predictable from their components, that some components are preferred to others in perception and that emergent properties can be discovered or explained using compositional models. Thus, compositionality is a powerful approach for understanding how whole objects relate to their parts.

## Graphical abstract

Can whole objects be understood in terms of their parts? Although this is a fundamental question for object perception, it has been difficult to answer because there are no unique decompositions of whole objects into parts, and any attempt to enumerate features can lead to a combinatorial explosion. Here, I review a novel approach using which we have shown that whole objects are surprisingly predictable from their components and that emergent properties be discovered or explained using these compositional models.

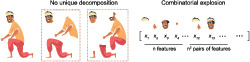

## 1 INTRODUCTION

**FIGURE 1:**
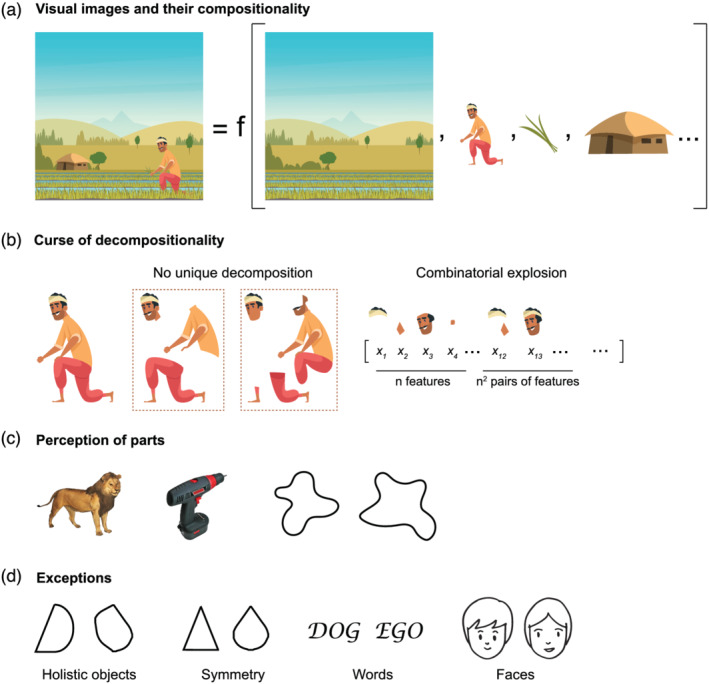
Visual objects and compositionality. (a) When we see this image, we see it as containing distinct components: the scene, the farmer, the rice plant, the house, and so on. Formally, we can define a representation to be compositional if it is a simple function of some components. Describing this image as a farmer holding a rice plant, sitting in a rice field near his house with mountains in the background is a useful compositional representation because the components—farmer, kneeling, house, and so on—contain collections of pixels that repeat in many other images with minor modifications. Describing this image as a collection of pixels is a compositional representation but not useful since the individual pixels are tiny and do not repeat across images. *[Image credit: Vecteezy.com]*. (b) Attempting to identify the parts or features that drive the response to an object leads to several fundamental problems, which we collectively denote as the ‘*curse of decompositionality*’. This is illustrated for a silhouette image although similar arguments apply to natural images. First, there is no unique way to enumerate features. Second, there is no common dictionary of features across all shapes. Third, object responses could be driven not only by single features but also pairs of features, triples, and so on—leading to a combinatorial explosion of possible predictors. (c) In perception, many objects are readily seen as containing parts. This is true for real‐world objects like the lion and drill (*left*), which are seen as containing distinct, functional parts. It is also true for abstract silhouettes (*right*), which are seen as containing protrusions. *[Image credits: Bank of Standardized Stimuli (BOSS), Vecteezy.com].* (d) Any approach to understand objects using their parts must also account for exceptions, some of which are depicted here. *Left to right*: Some ‘holistic’ objects do not seem to contain any parts. Some salient properties like symmetry cannot be attributed to any single part. Some objects, like words and faces, seem to be ‘seen as a whole’ without the experience of processing them part by part. *[Image credit: Vecteezy.com]*

Look at the idyllic image in Figure 1a, and we readily see a farmer standing a rice field holding a rice plant with his house and mountains in the background. That is, we see the visual image as made of distinct components. Some of these components are objects, some are scenes and others are relations between objects (see Box 1 for definitions). This illustrates the fundamental question of compositionality: does our visual system represent images using its components? Formally, we can define a compositional representation as one in which responses to an image can be described as a sum or product of distinct, non‐overlapping components. Conversely, a non‐compositional or holistic representation is one in which the responses to an image cannot be described as a simple sum or product of the components. For example, neural responses in higher visual areas are non‐compositional in terms of pixels since they cannot be explained as a sum or product of the responses to single pixels. By contrast, the neural response in higher visual areas might be compositional in terms of components such as the farmer, rice, field and house, since it can potentially be predicted as the sum or product of these distinct components. Thus, understanding whether a given object representation is compositional, and identifying the underlying components that make it so, is an important insight into any visual system.

In biological vision, answering this question can help elucidate how high‐level vision is organized and help treat the disorders of high‐level vision (Barton, 2011; King et al., 2017; Kubilius et al., 2015). Compositionality is similar in many ways with proposals that high‐level vision makes relevant information easy to decode through untangling (DiCarlo et al., 2012; DiCarlo & Cox, 2007). However, they are different in that compositionality refers to the encoding of the image using components, whereas untangling refers to the complementary question of decoding of the relevant properties.

In machine vision, understanding compositionality can help understand how complex machine vision systems work (Bau et al., 2020; Stone et al., 2017). Today, all we know about deep networks is that they represent images as feature vectors, but this description does not promote a deeper understanding of these networks, or reveal the principles governing their representations. Comparing compositionality in brains and machines could elucidate why human vision remains superior to machine vision despite impressive progress made recently (Jacob et al., 2021; Katti et al., 2017; Peters & Kriegeskorte, 2021; Serre, 2019), and eventually pave the way to better machine vision. Understanding the compositionality of visual systems is therefore a question of fundamental and practical significance.

Here, I summarize the difficulties with answering this question, briefly review previous approaches, describe experimental approaches to understand object representations and finally propose an approach to tackle these issues that we have found fruitful in a number of studies.

BOX 1: Definitions

- *Feature*: Any measurable property in an image regardless of functional significance (e.g., in Figure 1a: the V shaped intersection between the mountains, the L‐shaped edge formed between the farmer's back and the field, chin curvature, angle of left leg and conformation of right hand).
- *Representation*: A multidimensional vector representing an image—these could be a set of image features, or a collection of channel activations to an image.
- *Compositionality*: The property by which an image is represented as a simple function (e.g., sum or product) of a set of constituent components. For a compositional representation to be useful, the components must each encompass many pixels and must repeat in many images.
- *Components*: Features that make up a compositional representation (e.g., in Figure 1a—the farmer, rice plant, field, house, spatial relations such as ‘holding’, ‘sitting’, etc.).
- *Parts*: Features that are perceived as making up an object (e.g., in Figure 1a, hands and legs as parts of the farmer's body). While parts are likely to be components in a compositional representation, all components in an image may not be perceived as parts.

## 2 THE CURSE OF DECOMPOSITIONALITY

Understanding which components of an image drive neurons or behaviour is extremely difficult for several reasons, which I collectively denote as the *curse of decompositionality*. First, there is no unique way to divide an object into features, as depicted in Figure 1b: the same object can be broken down in many ways. Cutting object images into parts can be problematic because the new feature introduced by the cut could complicate any effort to relate the cut parts to the whole. Some features are more important than others: for instance, convex protrusions are more salient in perception (Bertamini & Wagemans, 2013; Cohen et al., 2005; Fantoni et al., 2005; Todd & Petrov, 2022) and have been used to model neural responses in high‐level visual areas (Brincat & Connor, 2004; Yamane et al., 2008). However, since objects can be broken down in many possible ways, even the minimal or critical set of features also becomes non‐unique as a result, whether in biological neurons (Bashivan et al., 2019; Kobatake & Tanaka, 1994; Ullman et al., 2016) or for that matter, in deep networks (Bau et al., 2020; Lapuschkin et al., 2019).

Second, there is no common dictionary for object parts or features. Rather, there have been a large diversity of approaches to represent object shape, as summarized elegantly in a recent review (Todd & Petrov, 2022). Some influential theories include recognition by components (Biederman, 1987), according to which all visual objects are made of a common dictionary of parts called geons, which can be recognized in a view‐invariant fashion (Biederman & Cooper, 1991; Biederman & Gerhardstein, 1993, 1995). An alternative idea is shape skeletons (Blum, 1973; Feldman & Singh, 2006), which are reduced object descriptions that could enable recognition by removing accidental variations from the image. These representations have been found to explain neural and behavioural responses (Ayzenberg et al., 2022; Firestone & Scholl, 2014; Hung et al., 2012). Nonetheless, it is appealing to consider whether our visual perception has an inherent grammar to its representations (Cavanagh, 2021).

Third, and most importantly, enumerating features leads to a combinatorial explosion of possibilities (Figure 1b), because if there are *n* possible features that could drive the response to an object, then there are *n* ^2^ possible pairs of features that could also drive its response, *n* ^3^ triples of features, and so on. Finding the unique subset of features that might drive the response becomes computationally intensive because of an explosion of possible subsets. Evaluating these models also becomes increasingly difficult because of the large demands of experimental data required to fit them. Perhaps recognizing this conundrum, many recent approaches to explaining neural or behavioural responses to objects have compared models containing differing sets of features to identify which model better explains the observed data (Peters & Kriegeskorte, 2021; Serre, 2019). However comparing model fits can be tricky because complex models automatically explain more variation in any data, and assessing model complexity is not straightforward in some cases, such as for deep neural networks. This problem is frequently addressed by cross‐validation, that is, by training several models on a subset of the data and comparing their performance on held‐out test data (Duda et al., 2001). Intuitively, this would work only if the test data are sufficiently different from the training data (Shen et al., 2021). However, whether this is so is not always clear: for instance, training models on one set of natural images and testing them on another set of natural images might not avoid overfitting if both sets are ‘close’ in the underlying representation. A deeper problem with models today is that, given their complexity and large number of parameters, their excellent agreement with neural data fails to be conceptually insightful. One could argue of course that prediction is all that matters and is equivalent to understanding, but there is a long arc of history in science where accurate prediction is a distinct phase that often precedes the discovery of deeper conceptual insights (Ajemian & Hogan, 2010; Fetz, 1992). For instance, a seminal development in physics was the accurate measurements and predictions of planetary motion made by Tycho Brahe, for which the best predictions were based on the earth‐centred equant model. These predictions were eventually falsified using ‘out‐of‐distribution’ data by Johannes Kepler, leading to his discovery of the much simpler heliocentric model, and the laws of planetary motion (Ajemian & Hogan, 2010).

Finally, any effort to understand object features must also account for our perception of objects and their parts. Many natural and artificial objects seem to contain parts (Figure 1c). This process is visual and automatic, as revealed by elegant behavioural paradigms (Palmer, 1977; Xu & Singh, 2002). However there are plenty of exceptions as well (Figure 1d). Some objects are holistic, that is, do not seem to contain parts (Figure 1d, *left panel*). Others have emergent properties (Wagemans et al., 2012). One example is symmetry, a property of the whole that is not present in any single part of an object (Figure 1d, *middle panel*). Yet other objects, such as words and faces, seem to be recognized at a glance without explicit recognition of the parts (Figure 1d, *right panel*), suggesting that they may be processed as a whole rather than as parts. Evidence in favour of this account comes from the slower matching of parts when isolated than when shown in context (Piepers & Robbins, 2012; Richler et al., 2012; Tanaka & Farah, 1993; Wong et al., 2011). However, these observations are also consistent with models based on parallel activation of parts (Fifić & Townsend, 2010).

## 3 EXPERIMENTAL APPROACHES TO STUDY OBJECT REPRESENTATIONS

**FIGURE 2:**
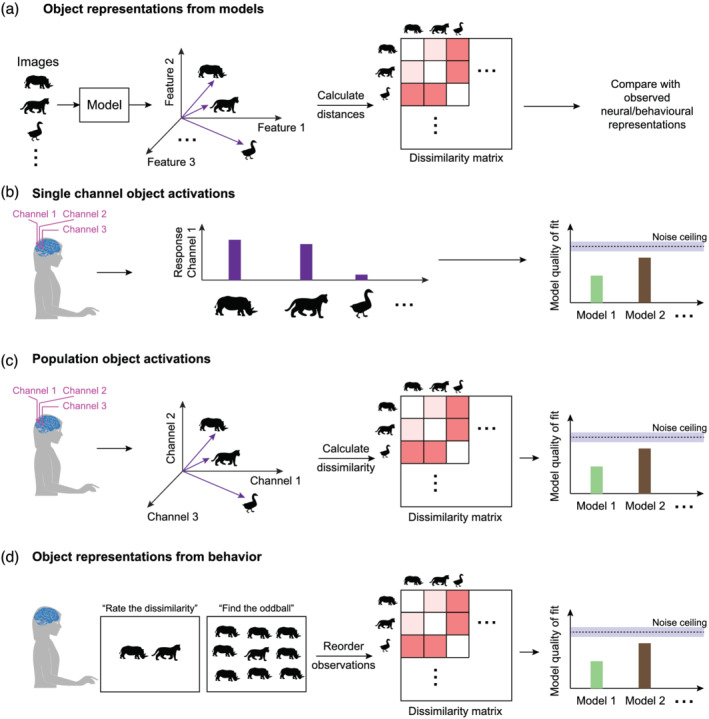
Approaches to study object representations. (a) *Object representations from models.* Here, each image is given as input to a model (image feature models, deep networks), which returns a feature vector. These feature vectors are used to construct pair‐wise dissimilarities between all images. These dissimilarities are compared with observed neural dissimilarities. (b) *Single channel object activations.* Here, neural responses (single unit, EEG, MEG, fMRI, etc.) of each channel are fit to quantitative models based on the stimuli. The noise ceiling represents an upper bound on model fits estimated based on noise levels in the neural responses *[Brain image credit: Macauley Breault, SciDraw.io].* (c) *Population object activations.* Here, neural responses across multiple channels are considered as a multidimensional vector, and pair‐wise dissimilarities are calculated between each pair of stimuli. The resulting dissimilarity matrix (*middle*) is compared against quantitative models based on the stimuli (*right*). The noise ceiling represents an upper bound on model fits estimated based on noise in the measured dissimilarities. (d) *Object representations from behaviour.* Here, dissimilarity between pairs of objects is directly measured from participants using dissimilarity rating tasks or visual search tasks, and these pair‐wise measurements can be used to construct a dissimilarity matrix.

To summarize, understanding the features or parts that determine the whole object response has been difficult due to conceptual challenges with identifying the underlying features. These challenges are common to both neural and behavioural data, but it is worthwhile at this point to review the experimental methods used to study object representations. These can be grouped into three broad categories, as summarized in Figure 2.

Current approaches to study object representations all involve the use of computational models, which we summarize briefly (Figure 2a). The input to a computational model is typically the image (or sometimes, specific image properties), and its output is a vector of numbers that represent the features extracted by the model. For example, the response of the 10th layer of a deep network would be a vector comprising the activations of all 1000 units in that layer. These activations can be used directly as predictors of the neural response. Alternatively, the response to pairs of images can be converted into pairwise dissimilarities (Figure 2a) that are then compared with behavioural or neural dissimilarities, as detailed below.

The first approach is to characterize single channel activations evoked by objects. These activations could be single unit activity in neurophysiological recordings, single channel electrical responses from scalp recordings or single voxel activations in brain imaging (Figure 2b). The goal of such efforts is to measure the variation in single channel activations across objects (Figure 2b, *middle panel*) and predict/understand them using computational models (Figure 2b, *right panel*). These model fits are typically compared against a noise ceiling, which is an upper bound for model performance based on noise in the observed data. Recent examples of this approach include models of neurons in the inferior temporal cortex (Bashivan et al., 2019; Chang et al., 2021; Higgins et al., 2021; Owaki et al., 2018; Ponce et al., 2019; Yamins & DiCarlo, 2016), models of single voxel tuning in fMRI (Ratan Murty et al., 2021).

The second approach is closely related to the first since it uses the same data but is conceptually different in its focus on the ensemble as a whole rather than single channels (Figure 2b). Here, the activation evoked by objects across all channels is taken as a multidimensional vector (Figure 2c, *left panel*), and pairwise dissimilarities between objects are calculated using a suitable distance metric (Figure 2c, *middle panel*). These pairwise dissimilarities capture the overall structure of the population representation rather than the idiosyncrasies of individual channels. On the other hand, they may miss important finer details of encoding in smaller subsets of neurons that could guide behaviour. These pairwise dissimilarities are typically compared to pairwise dissimilarities calculated from computational models (Figure 2c, *right panel*). These model fits are compared against a noise ceiling as before. This approach has become extremely popular because it allows for comparing object representations across experimental modalities by converting them all into the common currency of dissimilarity (Kriegeskorte et al., 2008). However, a disadvantage of this approach is that it can complicate statistical testing since the underlying dissimilarities are no longer statistically independent (Nili et al., 2014; Walther et al., 2015). This is because a large number of pairwise dissimilarities are being calculated from a small number of multivariate object responses. Recent examples of this approach include comparisons of object representations in the brain with behaviour (Jozwik et al., 2016; Mur et al., 2013; Proklova et al., 2016; Rajalingham et al., 2018), or both neural and behavioural representations with computational models including deep networks (Jacob et al., 2021; Pramod & Arun, 2022; Rajalingham et al., 2018; Ratan Murty et al., 2021; Storrs et al., 2021).

The third approach to study object representations comes from behavioural experiments (Figure 2d). In the classic approach, participants are asked to rate the dissimilarity between a pair of objects (Attneave, 1950; Dunn, 1983; Shepard, 1964; Tversky, 1977; Tversky & Gati, 1982). This is a complex task because rating perceived dissimilarity involves setting up an internal scale and mapping it to a numeric value, and because participants could use visual, verbal or semantic similarity to make a judgement (Torgerson, 1965). Dissimilarity ratings can also be easily biased or subverted by participants since there is no objective measure of performance.

We have recently used visual search as an alternative method to measure perceptual dissimilarity. In contrast to dissimilarity ratings, visual search is a natural task that is easy to understand and has an objective measure of performance. In this paradigm, participants have to simply locate an oddball target (Figure 2d, *left panel*). We have found across multiple studies that the inverse of the response time is an insightful measure for visual search. It not only satisfies the conditions for a mathematical distance metric (Arun, 2012) but has a straightforward physical interpretation. Since response times in visual search are thought to be due to an accumulation of evidence to threshold, the inverse of response time is an estimate of this accumulating signal. Thus, the inverse of search time can be interpreted as the underlying dissimilarity signal that drives visual search (Arun, 2012; Sunder & Arun, 2016).

Once these pair‐wise dissimilarities are collected through rating or search experiments, they can be compiled into a dissimilarity matrix (Figure 2d, *middle panel*) and compared with computational models or even neural dissimilarities (Figure 2d, *right panel*). Recent examples of this approach include comparisons of human similarity judgments with computational models (Hebart et al., 2020; Pramod & Arun, 2022).

## 4 THE BLESSING OF COMPOSITIONALITY

Current approaches to understanding object representations involve measuring neural or behavioural responses and explaining them using complex computational models, often deep neural networks. The advantage of this approach is that we have image‐computable models that can explain neural and behavioural responses to an object and that can be used as a testbed for preliminary investigations. The disadvantage of this approach is that, because these models are complex and uninterpretable, their excellent fits to the data do not offer any conceptual insights into object representations.

**FIGURE 3:**
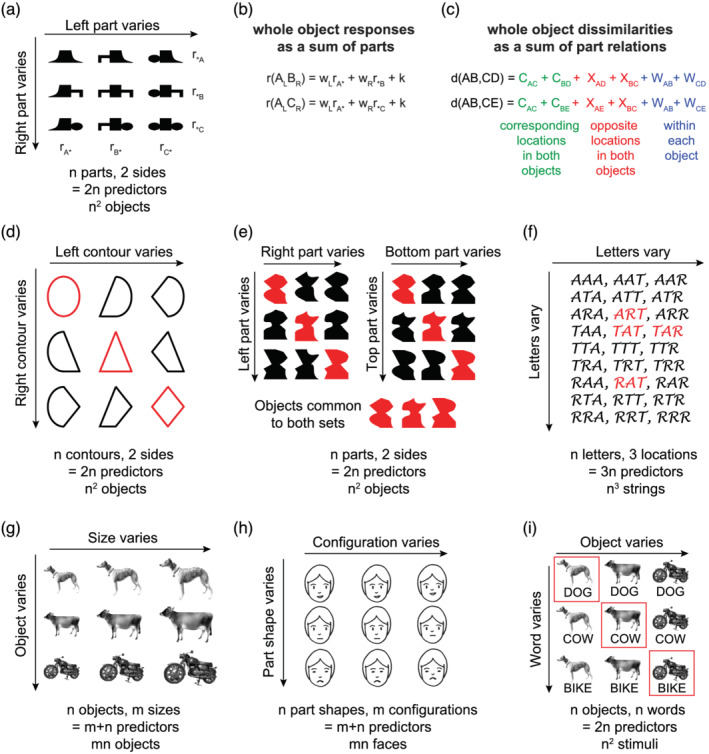
Blessing of compositionality. Creating a large number of objects using a small number of pre‐defined parts has many advantages, primarily because it allows defining parts‐based models that can be used to explain whole objects. A few possibilities are listed below. (a) A set of *n* ^2^ objects created by combining the same *n* contours on the left and right sides (adapted from Pramod & Arun, 2016). This design enables asking whether neural or perceptual responses to the *n* ^2^ objects can be predicted using the 2*n* predictors corresponding to the left and right contours. (b) If neural responses are measured experimentally for all the *n* ^2^ objects, then whole object responses can be predicted using the parts as follows: For each part A, calculate its contribution on the left side as the average response to all objects with this part on the left, denoted as $r_{A*}$. Likewise, on the right side, its contribution is $r_{*A}$. Then the response to the whole object AB is simply a weighted sum of part A on the left and part B on the right. This approach works because there are many objects containing the same part, allowing us to estimate its unique contribution (Pramod & Arun, 2018; Sripati & Olson, 2010). (c) If dissimilarities are measured experimentally for all possible pairs of objects, then for 49 objects created from seven parts, we would have ^49^C_2_ = 1176 experimentally measured dissimilarities. For each pair of objects (AB,CD), we can predict the net dissimilarity as a sum of all possible part‐part relations, which can be grouped into three types: part relations at corresponding locations in the two objects (i.e., A‐C & B‐D), part relations at opposite locations in the two objects (i.e., A‐D & B‐C) and part relations within each object (i.e., A‐B & C‐D). This model works because the same part relation (e.g., A‐C) is present in many dissimilarity measurements (e.g., AB‐CD & AB‐CE). From Pramod and Arun (2016). (d) Example set of holistic objects created by combining contours. Many resulting objects do not seem to contain parts, yet their dissimilarities can be predicted using part relations. This design also enables us to ask whether symmetric objects (such as those highlighted in red) show any deviations from the parts‐based models (see Pramod & Arun, 2018). (e) Two sets of *n* ^2^ objects created with left/right parts varying, or top/bottom parts varying, but designed such that the diagonal items (highlighted in red) are identical in both sets. This design allows asking whether the representation of the diagonal items is better explained using the top/bottom parts or the left/right parts (see Pramod & Arun, 2016). (f) Three‐letter words created by combining *n* letters at three locations, which enable us to ask if their representations can be understood using single letters. It also enables us to ask whether real words (highlighted in red) show any deviations from the letter‐based models (see Agrawal et al., 2020). (g) A set of objects created at many possible sizes, enabling us to ask how object identity and invariant attributes combine. Here, *m* + *n* predictors corresponding to object identity and size can be used to explain the responses to *mn* objects (see Ratan Murty & Arun, 2018). (h) A set of faces created by varying face parts and configurations in all possible combination. Here, *m* + *n* predictors corresponding to *n* part shapes and *m* configurations can be used to explain the responses to *mn* faces, allowing us to ask how shape and configuration features combine, and test for holistic properties by studying model errors. (i) A set of *n* object pictures are combined in all possible ways with *n* words, to create *n* ^2^ possible images. This design enables us to ask how picture and word information combine in general and whether the congruent entries (highlighted in red), which represent matching pictures and words, show any deviations from the component‐based models.

Here I describe a complementary approach that has proved fruitful in our investigations, which I refer to as the *blessing of compositionality*. The key design principle is summarized in Figure 3a. Here, we took a fixed number of *n* contours placed either on the left or right sides, and combined them in all possible ways to create *n* ^*2*^ possible objects. The advantage of this design is that a large number of objects is now created from a small number of parts. For example, a set of 49 objects can be created by combining seven parts on either side, leading to 49 responses that have to be predicted using only 14 predictors (7 contours × 2 sides). Note that presenting any isolated feature separately can be problematic since isolating it can create edge artefacts where it is cut from the whole object (e.g., the body parts in Figure 1b). By contrast, this approach allows estimating the contribution of the predictors without ever directly presenting them as stimuli.

We have used such models to explain the neural responses to objects as a sum of parts (Katti & Arun, 2022; Pramod & Arun, 2018; Sripati & Olson, 2010). The key idea is shown in Figure 3b. For part A, we simply take its average contribution on the left side as the average response to all objects with part A on the left side as $r_{A*}$. Likewise, the contribution of part A on the right side is simply the average response to all objects with A on the right, denoted as $r_{*A}$. We can then predict the net response to a two‐part object AB as the weighted sum of the corresponding average responses $w_{L}r_{A*}+w_{R}r_{*B}$. This approach works because the same part $r_{*A}$ is present in multiple whole objects (e.g., in objects AB and AC; see Figure 3b). A similar approach can be used to estimate multiplicative models where the net response to AB is given as $r_{\mathrm{AB}}=r_{A*}r_{*B}$ (Katti & Arun, 2022; Ratan Murty & Arun, 2018).

This approach can also be used for whole object dissimilarities, measured in behaviour or using neural population recordings. If we were to measure all pairwise dissimilarities between objects, for 49 whole objects, this would mean ^49^C_2_ = 1176 measurements that can be explained essentially using only ^7^C_2_ = 21 part‐part dissimilarities (here, ^*n*^C_*k*_ is shorthand for ‘*n* choose *k*’, or the number of ways of choosing *k* unique items from a set of *n* items). Here, we assume that the net dissimilarity between two objects AB & CD can be described using all pair‐wise part‐part relations. These part relations can be divided into three types: relations between parts at corresponding locations in both objects (A‐C & B‐D), relations between parts at opposite locations in both objects (A‐D & B‐C) and relations between parts within each object (A‐B & C‐D), as depicted in Figure 3c. This approach works because the same part‐part relation (e.g., A‐C) is present in multiple dissimilarities (e.g., in AB‐CD & AB‐CE), and the unknown part relations can be easily estimated using linear regression. We have used such models to explain object dissimilarities using part dissimilarities in a variety of objects (Pramod & Arun, 2016). Likewise, hierarchical stimulus representations can be understood as a linear sum of global and local shape differences (Jacob & Arun, 2020). Together, these results suggest that whole object representations can indeed be understood in terms of their parts.

Another major advantage to this design is that it results in the creation of special combinations, such as symmetric objects, which are located along the main diagonal of the stimulus matrix (Figure 3d). This enables us to ask whether models that are based on simple summation of parts show systematic deviations for these special combinations. Remarkably, symmetric objects showed no such systematic deviations. Instead, we found that symmetric objects are distinctive in perception because their response is produced through repeated summation of the same part (Pramod & Arun, 2018). By contrast, we found systematic errors in models that used only length and width changes to explain dissimilarities between rectangles, suggesting nonlinear integration. However, including an additional feature, aspect ratio, fully explained the data using linear summation (Pramod & Arun, 2014). We speculate that this approach can be used to detect novel holistic properties by looking for systematic error in compositional models (Wagemans et al., 2012).

This compositional approach can also be used to distinguish between possible part decompositions. In a separate experiment, we investigated whether the visual system preferentially decomposes objects into one set of parts rather than another set (Pramod & Arun, 2016). To this end, we created two compositional sets of objects, one in which the left and right parts vary (Figure 3b, *left*) and one in which the top and bottom parts vary (Figure 3e, *right*). We designed these two sets such that the diagonal entries resulted in identical shapes. For each of these two sets, we measured all pairwise dissimilarities between objects in the set and asked if they could be explained by part‐part dissimilarities. Our hypothesis was that, if the common objects were preferentially broken down into top and bottom parts in our perception, then they should be explained better by the model based on top‐bottom parts compared to the model based on left–right parts. We confirmed that this was indeed the case (Pramod & Arun, 2016).

This compositional approach can also be used to understand visual word representations (Agrawal et al., 2019, 2020). For example, we can measure pairwise dissimilarities between letter strings created by placing *n* letters at multiple locations. This can lead to a large number of possibilities: for example, placing 10 letters at 3 locations results in 1000 possible three‐letter strings (Figure 3f). However, the underlying models have only 30 parameters (10 letters × 3 locations), so in practice a smaller subset of three‐letter strings can be sampled to fit the data. A similar argument works for string dissimilarities as well. Although there are ^1000^C_2_ = 499,500 possible pairwise dissimilarities, the underlying part‐part dissimilarities are only ^10^C_2_ = 45, which means that a smaller subset of pairwise dissimilarities need to be sampled to fit part‐based models. An important feature of this design is that some letter strings are special because they are real words. If visual words are detected holistically using whole‐word detectors, then we reasoned that they should show larger model errors since the part‐based models are agnostic to these special combinations. Surprisingly, this was not the case: we did not observe larger model errors for real words compared to other letter strings, suggesting that real word representations are not holistic but part‐based (Agrawal et al., 2020). Even more interestingly, we compared letter and letter‐pair (bigram) representations in readers and non‐readers of two distinct Indian languages, and found equally good model fits for bigram representations in readers compared to non‐readers. This is again contrary to the greater model error expected if readers had formed bigram detectors as a result of learning to read (Agrawal et al., 2019). This led to a surprising conclusion: reading is facilitated not by whole‐object detectors but by more efficient parsing of bigrams. This was evidenced by weaker within‐bigram interactions in readers, which predicted their reading fluency (Agrawal et al., 2019). More recently, we have found that letter interactions in upright (not inverted) bigrams explained reading fluency variations in children even after accounting for other language based measures (Agrawal et al., 2022).

The compositional approach can also be extended to study disparate attributes. Consider for example the objects shown in Figure 3g. Taking *n* = 20 objects at *m* = 10 sizes leads to a set of 200 stimuli that can be potentially explained by a set of only 30 predictors (20 objects + 10 sizes), provided size and object identity are separably encoded. This separable encoding can be additive or multiplicative: that is, the response could be the sum or product of object identity and size tuning. Remarkably, we have found that single neurons in high‐level visual areas encode object identity and invariant attributes (size/position/viewpoint) multiplicatively, but combined object parts additively (Ratan Murty & Arun, 2018). In a more recent study, we have shown that single neurons encode distorted letters multiplicatively but letter strings additively, and that this separable encoding can lead to perfect decoding of distorted letters (Katti & Arun, 2022). We have observed similar results in behaviour as well: shape differences combine linearly with texture as well as with global configurations (Pramod & Arun, 2016).

As the above examples demonstrate, compositional designs can be a powerful approach to understanding a variety of object representations. We illustrate the power of this approach by offering up two example designs that can be readily tested in future studies. In Figure 3h, we have created a large set of faces by combining part shape and part configuration in a combinatorial fashion. Measuring neural or behavioural responses to these shapes can reveal whether these two properties are separably encoded. In Figure 3i, we created a large set of picture‐word pairs by combining a small set of pictures and their corresponding words in all possible ways. Measuring neural or behavioural responses to this set could reveal how pictures and words are encoded jointly, and evaluating the special combinations (congruent picture–word pairs) could reveal where pictures and words are matched in the brain.

## 5 RELATION TO OTHER APPROACHES

Our proposed approach is similar to classic studies of separability of receptive field properties in sensory neurons. For example, multiplicative separability has been observed in gain fields in parietal neurons (Salinas & Thier, 2000), in auditory cortex (Peña & Konishi, 2001) and in spatiotemporal receptive fields of neurons in early visual cortex (DeAngelis et al., 1993; Grunewald & Skoumbourdis, 2004; Smolyanskaya et al., 2013). However, we have extended these ideas to include additive as well as multiplicative separability (Katti & Arun, 2022) and to include abstract properties such as object identity and image attributes (Ratan Murty & Arun, 2018), and to include both neural and behavioural data.

Our proposed approach is also concordant with recent studies that have used similar designs to study object representations. There is now considerable evidence that the neural responses to multiple objects is accurately predicted as a linear sum of the individual object responses (Agam et al., 2010; Baeck et al., 2013; Kaiser et al., 2014; MacEvoy & Epstein, 2009; Reddy et al., 2009; Zoccolan et al., 2005). Interestingly, a recent study showed that it can be insightful to study deviations from compositionality: objects in familiar configurations showed greater deviations from part summation compared to objects in unfamiliar configurations (Kaiser & Peelen, 2018). Thus, parts in familiar configurations might behave like unitary wholes, and it will be interesting how such deviations develop with experience. However, all these studies use separated object pairs, where the individual objects can be easily isolated for testing. By contrast, testing whole objects like bodies is complicated by the fact that separating them into parts like face, torso, hands etc leads to additional features near the part cuts (as seen in Figure 1d). Indeed, a recent study has reported that whole body responses cannot be explained using the isolated parts, but this could well be due to the additional cut features present in the isolated parts (Brandman & Yovel, 2016). Our approach circumvents this problem by indirectly estimating the component contributions from the compositional design.

More broadly, our proposed approach falls into the long tradition of systematic feature manipulations that are widely used to understand object and face perception. However, most studies have focused on characterizing or comparing perceptual or neural sensitivity to single feature manipulations, and have only rarely investigated how features combine. By contrast, the approach I have proposed puts the focus explicitly on understanding how object features or components combine, with the provision to detect emergent properties as deviations from compositionality.

## 6 LIMITATIONS OF COMPOSITIONAL DESIGNS

The approach of using compositional experimental designs has many advantages as detailed above but also has several limitations. First, it relies on the creation of a large number of arbitrary combinations. When the components are many, it can be impractical to sample all possible combinations, and we will need novel approaches for optimal sampling of stimuli or pairwise distances that enable accurate estimation of component‐based models (Agrawal et al., 2020). Some combinations may also be impossible to create: for instance, no object can have too many concave surfaces. Yet other combinations can result in novel features that can complicate the interpretation of component‐based models: for instance, juxtaposing arbitrary contours on the left and right side of an object could create unique and novel intersections whose response cannot be explained using component‐based models. We have overcome this limitation by ensuring that contours always terminate in the same two points (Figure 3a). Second, while synthesizing stimuli in compositional designs is easy, analysing natural images or objects into components remains non‐trivial: it is not altogether clear that every natural image can be described as clearly in terms of components as the farmer image in Figure 1a. Doing so would require identifying unique, repeating components in natural images and compiling collections of natural images with these components occurring in enough combinations to fit compositional models. Such approaches can be potentially tested on speech signals where the underlying components are defined more clearly (vowels and consonants), and then extended to visual images where the underlying components are less clear. Third, while compositional designs implicitly assume discrete sampling of stimuli, this is only a matter of convenience, since the same approach could be extended to continuously sampled features, provided both features are sampled uniformly.

## 7 CONCLUSIONS AND OPEN QUESTIONS

Here, I have described a compositional approach to studying object vision, in which a large number of objects are created using a small number of components. This design allows for formulating component‐based models that can explain neural or behavioural responses to whole objects. Systematic errors in these compositional models can be used to discover novel, emergent properties of objects that cannot be explained using their parts. I propose that using compositional experimental designs can lead to interesting and important insights into object representations of both biological vision and machine vision systems, as well as other broad questions about the nature of sensory representations and how they are learned (Box 2).

BOX 2: Open questions for compositionality

- Is compositionality a fundamental principle for high‐level perception? Does this motif repeat in other brain regions? In other sensory modalities?
- What are the possible computations (sum, product, other) that are used to combine components of a compositional representation?
- How does compositionality in high‐level sensory areas compare with compositionality in low‐level sensory areas? Are the components different? How does one compositional representation transform into another?
- Can compositionality be learned through experience? What kind of sensory experience is sufficient for learning compositional representations?
- Can compositionality be learned faster with specific types of priors, such as constraints/principles embedded into neural networks?
- Can enforcing compositionality during training lead to better performance in deep networks? Are such principles observable during brain development?

## CONFLICT OF INTEREST

I declare no conflict of interest.

## AUTHOR CONTRIBUTIONS

SPA wrote the manuscript.

### PEER REVIEW

The peer review history for this article is available at https://publons.com/publon/10.1111/ejn.15746.

## Data Availability

Not applicable since this is an invited review.

## SP ARUN'S ACHIEVEMENTS AND REFLECTIONS ON UNDERTAKING A CAREER AS A NEUROSCIENTIST

**S. P. Arun** I grew up in Hyderabad, India and obtained my undergraduate degree in Electrical Engineering from the Indian Institute of Technology, Bombay. After that, I went to the United States to complete my MSE and PhD in Electrical and Computer Engineering at the Johns Hopkins University, where I made my transition to neuroscience. My thesis advisors were Profs. Kenneth Johnson and Andreas Andreou, and I studied the somatosensory system. During my postdoctoral training with Prof. Carl Olson at Carnegie Mellon University, I began to study object representations in high‐level vision. In 2010, I moved back to India as an Assistant Professor at the newly formed Centre for Neuroscience at the Indian Institute of Science, Bangalore. My lab studies object representations in high‐level vision using a combination of human behaviour/brain imaging, monkey behaviour/neurophysiology and computational modelling.

## What led you to become a neuroscientist?

I was always fascinated by science fiction and robotics, so when I started a PhD in Electrical and Computer Engineering at Johns Hopkins, I started enquiring about courses in artificial intelligence and robotics. But, my friend, Faisal Beg, got me thinking when he asked me why I was not interested in *natural intelligence*, that is, the brain. He referred me to talk to a neuroscientist, Prof. Ken Johnson, an electrical engineer himself, who offered that I could join his lab for my PhD. I would never have predicted becoming a neuroscientist, but today I cannot imagine not being one!

## What external influences or mentors had a significant impact on your scientific career?

I've had lots of inspiration. A major influence was from my parents, who are both scientists: I learned a lot of hands‐on tinkering from my dad, and learned about the process of science from the experiences my mom would share with me about her own work, and the stories she told me about famous scientific discoveries, such as how Kepler discovered the laws of planetary motion.

I have also been extraordinarily fortunate to have mentors like my PhD advisor (Ken Johnson) and postdoctoral mentor (Carl Olson). Both were not only wonderful human beings, but also inspiring, rigorous scientists who thought deeply about the brain and how to get insights into sensation and perception. Importantly, they also gave me the freedom to explore without pressure or practical limitations. I have tried to do the same for my own students throughout.

## What area of neuroscience would you choose to pursue if you were currently at the beginning of your career? The same or a different topic?

I'd probably choose vision again but I find a lot of neuroscience pretty fascinating! At a close second, there is speech/language and then memory.

## What are the main challenges young neuroscientists face to build an independent and successful career in research? How did you overcome those?

The main challenge is to keep the focus on the core of science. It is all too easy to get distracted by setting up the lab, project planning, expenditure reports, grant writing, publications, impact factors, promotion/tenure, etc. All these activities can easily make you lose sight of the core purpose of whether you are really understanding the brain. It is also important to care and understand the people who work with you, and help them become better scientists. Another major challenge is giving priority to the family – you can never borrow too much time from family and put it into work. I try to maintain a regular schedule so that there's a daily work‐family life balance, and I try my best to disconnect from work once I get home. Of course, I am keenly aware of my own shortcomings in all these ideals! But it is useful to remember them to maintain a balanced perspective and have an ideal to aspire towards.

## How important do you consider the role of social medias in increasing the impact of your research? What platforms do you prefer to share your scientific work with the community? Why?

I believe it is our duty as scientists to communicate our findings to the public at large, since we are supported by public funds. It is also incredibly important to convey the process required to reach a scientific conclusion/result and not just the conclusion/result itself, which often just looks like information. Recently I have discovered Twitter (I am @sparuniisc) as a great means to find like‐minded people, interact with the broader community and of course share our findings with the public. I try to post a lay summary on twitter for each new study we publish, to communicate our findings with fellow scientists as well as lay readers interested in science.
